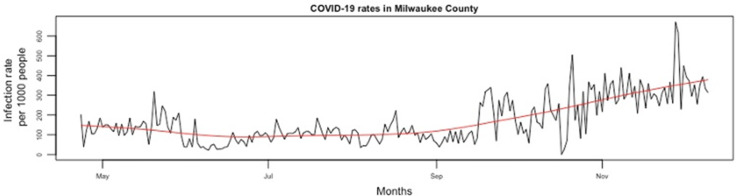# Predicting SARS-CoV-2 Asymptomatic Infection Rate of Inpatients: A Time Series Analysis

**DOI:** 10.1017/ash.2021.99

**Published:** 2021-07-29

**Authors:** Frida Rivera, Kwang Woo Ahn, L. Silvia Munoz-Price

## Abstract

**Background:** Asymptomatic SARS-CoV-2 infections play a crucial role in viral transmission. However, they are often difficult to identify given that widespread surveillance has not been the norm. We sought to determine whether COVID-19 rates reported at the county level could predict the positivity rates for SARS-CoV-2 among asymptomatic patients tested in a large academic health system. **Methods:** This observational study was conducted from April 23, 2020, to December 10, 2020, at Froedtert Health (FH) system, the largest academic health system in Wisconsin. On April 23, 2020, FH implemented SARS-CoV-2 surveillance among all consecutive admissions not suspected of COVID-19, all patients scheduled for elective procedures and deliveries, and all asymptomatic patients with known exposures. Samples were processed by the FH laboratory using molecular methods (RT-PCR). To obtain the daily number of newly confirmed COVID-19 cases in Milwaukee County, we accessed the Wisconsin Department of Health Services publicly available COVID-19 database. For the purpose of this study, COVID-19 rates were defined as the percentage of positive tests among all daily tests performed at the county level, while SARS-CoV-2 positivity rates were the percentage of positive tests among all daily surveillance tests performed at FH among asymptomatic patients. The association between COVID-19 rates in Milwaukee County and asymptomatic rates at FH were assessed using an autoregressive moving average time series analysis. To examine the association between these rates, we fitted a seventh-order autoregression for the residuals based on autocorrelation function and partial autocorrelation function plots of the residuals from linear regression. **Results:** From April 23, 2020, to December 10, 2020, there were 2,347 new asymptomatic infections detected at FH and 75,196 new COVID-19 cases reported in Milwaukee County. Figure 1 shows the time-series plot of asymptomatic SARS-CoV-2 positivity rates at FH and Figure 2 shows COVID-19 rates in Milwaukee County. As the COVID-19 rate in Milwaukee County increased by 1 unit, the asymptomatic infection rate in FH decreased by 0.024 unit (95% CI, −0.053 to 0.004; *P* = .095) after accounting for autocorrelation over time. Thus, there was no association between these rates. **Conclusions:** The positivity rates among asymptomatic patients at a large medical center were not predicted by the positivity rate at the county level. This finding suggests that the epidemiology at a county level may be determined by pockets in the population who may not interact, and thus not affect, the positivity rates among asymptomatic patients served by a hospital system within the county.

**Funding:** No

**Disclosures:** None

**Figure 1.**
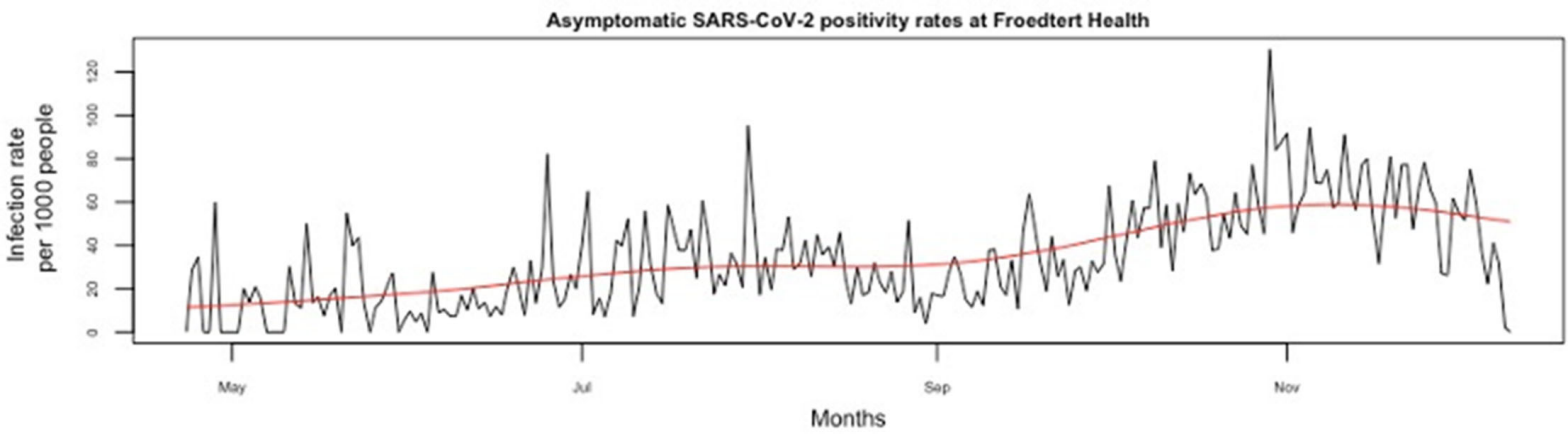

**Figure 2.**